# Interpreting the concordance statistic of a logistic regression model: relation to the variance and odds ratio of a continuous explanatory variable

**DOI:** 10.1186/1471-2288-12-82

**Published:** 2012-06-20

**Authors:** Peter C Austin, Ewout W Steyerberg

**Affiliations:** 1 Institute for Clinical Evaluative Sciences, G1 06, 2075 Bayview Avenue, Toronto, Ontario, M4N 3M5, Canada; 2 Department of Health Management, Policy and Evaluation, University of Toronto, Toronto, Canada; 3 Dalla Lana School of Public Health, University of Toronto, Toronto, Canada; 4 Department of Public Health, Erasmus Medical Centre, Rotterdam, The Netherlands

**Keywords:** Logistic regression, c-statistic, Area under the receiver operating characteristic curve, ROC curve, Discrimination, Regression model, Prediction, Predictive model, Predictive accuracy

## Abstract

**Background:**

When outcomes are binary, the c-statistic (equivalent to the area under the Receiver Operating Characteristic curve) is a standard measure of the predictive accuracy of a logistic regression model.

**Methods:**

An analytical expression was derived under the assumption that a continuous explanatory variable follows a normal distribution in those with and without the condition. We then conducted an extensive set of Monte Carlo simulations to examine whether the expressions derived under the assumption of binormality allowed for accurate prediction of the empirical c-statistic when the explanatory variable followed a normal distribution in the combined sample of those with and without the condition. We also examine the accuracy of the predicted c-statistic when the explanatory variable followed a gamma, log-normal or uniform distribution in combined sample of those with and without the condition.

**Results:**

Under the assumption of binormality with equality of variances, the c-statistic follows a standard normal cumulative distribution function with dependence on the product of the standard deviation of the normal components (reflecting more heterogeneity) and the log-odds ratio (reflecting larger effects). Under the assumption of binormality with unequal variances, the c-statistic follows a standard normal cumulative distribution function with dependence on the standardized difference of the explanatory variable in those with and without the condition. In our Monte Carlo simulations, we found that these expressions allowed for reasonably accurate prediction of the empirical c-statistic when the distribution of the explanatory variable was normal, gamma, log-normal, and uniform in the entire sample of those with and without the condition.

**Conclusions:**

The discriminative ability of a continuous explanatory variable cannot be judged by its odds ratio alone, but always needs to be considered in relation to the heterogeneity of the population.

## Background

Logistic regression models are frequently used to determine the association between a set of explanatory variables and a binary or dichotomous outcome variable. There are three primary reasons for fitting a logistic regression model: i) to determine the independent predictors of a binary outcome; ii) to determine the association between a specific variable and the probability of the occurrence of an outcome after adjusting for a set of other covariates; and iii) to predict the probability of the occurrence of a binary outcome given a specific vector of covariates. The third reason for fitting a logistic regression model occurs frequently in biomedical research, where researchers are interested in predicting the prognosis of individual patients [1].

Two key elements in assessing the performance of a fitted logistic regression model are the assessment of model calibration and model discrimination. Calibration refers to the agreement between observed outcomes and predictions, while discrimination refers to the ability of model predictions to discriminate between those with and those without the outcome [1,2]. The discriminative-ability of a logistic regression model is frequently assessed using the concordance (or c) statistic, a unitless index denoting the probability that a randomly selected subject who experienced the outcome will have a higher predicted probability of having the outcome occur compared to a randomly selected subject who did not experience the event. One can calculate the c-statistic by taking all possible pairs of subjects consisting of one subject who experienced the event of interest and one subject who did not experience the event of interest. The c-statistic is the proportion of such pairs in which the subject who experienced the event had a higher predicted probability of experiencing the event than the subject who did not experience the event [3]. The c-statistic can also be interpreted as the rank correlation between predicted probabilities of the outcome occurring and the observed response: it is equal to the Wilcoxon rank sum statistic for measuring the rank correlation between observed and predicted outcomes divided by the product of the number of subjects with the outcome or condition and the number of subjects without the outcome or condition [4,5]. It is also related to Somer’s D_*xy*_ rank correlation between the predicted probability of the occurrence of the outcome and the observed outcome: $D_{\mathrm{xy}}=2\left(c-0.5\right)$[3].

The discrimination of a logistic regression model can also be described by the area under the receiver operating characteristic (ROC) curve, often denoted by AUC [3]. Each value of the predicted probability of the occurrence of the outcome allows one to determine a threshold. For each possible threshold, one can dichotomize the predicted probabilities into those above and below the threshold. Subjects with a predicted probability below the threshold are classified as low risk, while those above the threshold are classified as high risk. One can then estimate the sensitivity and specificity of these classifications. The ROC curve is the plot of sensitivity vs. one minus specificity over all possible thresholds. The area under the ROC curve is equivalent to the c-statistic [4,5].

The relationship between the c-statistic of a logistic regression model and the regression coefficients and the variance-covariance of the explanatory variables has not been fully explored. The objective of the current paper was to examine the relationship between the c-statistic and the regression parameters and the variance of the explanatory variable in the case of a univariate logistic regression model. We first use mathematical derivations to explicitly derive the relationship between the c-statistic and the outcome odds ratio and the variance of the continuous explanatory variable under the assumption that the continuous explanatory variable follows a normal distribution in subjects with and without the outcome. Second, we use Monte Carlo simulations to examine this relationship in a more general setting.

### Mathematical derivation of the c-statistic under the assumption of binormality

Many derivations concerning the discrimination of different procedures require the assumption that the distribution of a continuous explanatory variable is normally distributed in those with the condition or outcome and also in those without the condition or outcome [4,6-9]. Thus, in each of the two populations (those with the condition or outcome and those without the outcome or condition), the explanatory variable is assumed to be normally distributed. Therefore there are two normal distributions: a normal distribution in those subjects with the condition or outcome and a normal distribution in those subjects without the condition or outcome. In the literature on statistical methods for diagnostic medicine, this assumption has been referred to as the binormality assumption [10].

Let Y denote the dichotomous response variable indicating the presence or absence of the outcome or condition of interest. Let X denote the continuous explanatory variable. We assume that X has means μ_A_ and μ_U_ and variances $\sigma_{A}^{2}$ and $\sigma_{U}^{2}$in the affected (Y = 1) and unaffected (Y = 0) populations, respectively. Finally, we assume that β is the log-odds ratio relating X to the dichotomous outcome Y: a one-unit increase in X results in a relative increase of the odds of the event occurring by exp(β). Finally, let $\Phi \left(\right)$ denote the standard normal cumulative distribution function. We let AUC denote the area under the ROC curve, which is equivalent to the c-statistic.

### General derivation: no restrictions on $\sigma_{A}^{2}$ and $\sigma_{U}^{2}$

We begin our derivation using a result derived by Zhou et al. [10]. Using the notation from Zhou et al., let $a=\frac{{\hat{\mu }}_{A}-{\hat{\mu }}_{U}}{{\hat{\sigma }}_{A}^{2}}$ and $b=\frac{{\hat{\sigma }}_{U}}{{\hat{\sigma }}_{A}}$. Then, $\text{AUC}=\Phi \left(\frac{a}{\sqrt{1+b^{2}}}\right)=\Phi \left(\frac{\frac{{\hat{\mu }}_{A}-{\hat{\mu }}_{U}}{{\hat{\sigma }}_{A}^{2}}}{\sqrt{1+{\left(\frac{{\hat{\sigma }}_{U}}{{\hat{\sigma }}_{A}}\right)}^{2}}}\right)=\Phi \left(\frac{{\hat{\mu }}_{A}-{\hat{\mu }}_{U}}{\sqrt{{\hat{\sigma }}_{A}^{2}+{\hat{\sigma }}_{U}^{2}}}\right)$, where Ф() denotes the cumulative normal distribution function. Thus, with no restrictions on the variances in the two groups, the c-statistic is a function of only the means and variances of the continuous explanatory variable in those affected and unaffected by the condition. The above expression can be rewritten as:

(1) $$\text{AUC}=\Phi \left(\frac{{\hat{\mu }}_{A}-{\hat{\mu }}_{U}}{\sqrt{{\hat{\sigma }}_{A}^{2}+{\hat{\sigma }}_{U}^{2}}}\right)=\Phi \left(\frac{{\hat{\mu }}_{A}-{\hat{\mu }}_{U}}{\sqrt{2}\sqrt{\frac{{\hat{\sigma }}_{A}^{2}+{\hat{\sigma }}_{U}^{2}}{2}}}\right)=\Phi \left(\frac{d}{\sqrt{2}}\right)$$

where *d* denotes the standardized difference or Cohen’s effect size [11-14]. The standardized difference is the difference in means in units of pooled standard deviation. Thus, the c-statistic is a function only of the difference in means between those affected and unaffected by the condition, in units of standard deviation. Since the standard normal distribution function is an increasing function, the c-statistic increases as the difference in the mean of the explanatory variable between those with and without the condition increases.

### Special case: $\sigma_{A}^{2}$ = $\sigma_{U}^{2}$

In the special case when the explanatory variable has the same variance in those affected and unaffected by the condition, one can simplify the above result using a result from discriminant analysis. Let σ^2^ denote the common variance of explanatory variable in the two groups. Then, the log-odds ratio relating the explanatory variable X to the log-odds of the occurrence of the condition has the following property: $\beta =\frac{\mu_{A}-\mu_{U}}{\sigma^{2}}$[15] (page 19). We then have that

(2) $$\text{AUC}=\Phi \left(\frac{{\hat{\mu }}_{A}-{\hat{\mu }}_{U}}{\sqrt{{\hat{\sigma }}_{A}^{2}+{\hat{\sigma }}_{U}^{2}}}\right)=\Phi \left(\frac{{\hat{\mu }}_{A}-{\hat{\mu }}_{U}}{\sqrt{2{\hat{\sigma }}^{2}}}\right)=\Phi \left(\frac{{\hat{\mu }}_{A}-{\hat{\mu }}_{U}}{\sqrt{2}\hat{\sigma }}\right)=\Phi \left(\frac{\hat{\sigma }}{\sqrt{2}}\frac{{\hat{\mu }}_{A}-{\hat{\mu }}_{U}}{{\hat{\sigma }}^{2}}\right)=\Phi \left(\frac{\hat{\sigma }\beta }{\sqrt{2}}\right)$$

Thus, when the explanatory variable is normally distributed in both those affected and unaffected by the condition, and furthermore has the same variance in both groups, then the c-statistic is a function of only the log-odds ratio relating the explanatory variable to the occurrence of the condition and the variance of the explanatory variable in each of the two groups. Since the standard normal distribution function is an increasing function, the c-statistic increases with increasing log-odds ratio relating the explanatory variable to the outcome and the standard deviation of the explanatory variable. Finally, the c-statistic is independent of the proportion of subjects with the condition.

### Accuracy of predicted c-statistic when the distribution of the explanatory variable is normal in the combined population of those with and without the condition

The analytic derivations in the previous section assumed that the explanatory variable is normally distributed in those with and without the condition. A potentially more realistic scenario is when the explanatory variable is normally distributed in the overall sample, rather than in those with and without the condition. In many such instances, it would be reasonable to expect the distribution of the explanatory variable to be approximately normally distributed in those with and without the condition. In this section we describe an extensive set of Monte Carlo simulations conducted to examine the accuracy of the predicted c-statistic derived under the assumption of binormality in the setting when the explanatory variable is normally distributed in the overall population.

### Monte Carlo simulations-methods

We simulated a continuous explanatory variable for each of 1,000 subjects from a normal distribution with mean 0 and standard deviation $\sigma_{\text{mc}}$: $x_{i}\sim N\left(0,\sigma_{\text{mc}}\right)$ for *i* =1, …, 1,000. We determined a linear predictor as follows: $\text{logit}\left(p_{i}\right)=\beta_{0}+\beta_{1}x_{i}$, where $p_{i}$ denotes the probability of a binary condition occurring. For each subject, we then randomly generated a binary condition from a Bernoulli distribution with subject-specific parameter $p_{i}$. We then fit a univariate logistic regression model (in which the binary condition was regressed on the continuous explanatory variable X) in the simulated dataset and estimated the c-statistic of the fitted model, which we refer to as the empirical c-statistic. We also determined the predicted c-statistic using the formulas (1) and (2) from Sections 2.1 and 2.2, respectively. To apply formula (1) from Section 2.1, we determined the mean and variance of the explanatory variable in those with and without the condition. To apply formula (2) from Section 2.2, we used the estimated regression coefficient (log-odds ratio) from the logistic regression model relating the explanatory variable to the presence of the condition and an estimate of the common variance of the explanatory variable in those with and without the condition. This estimate of the common variance was obtained as the variance in the combined sample of those with and without the condition. We repeated the above process 500 times. The mean empirical c-statistic along with the mean of the predicted c-statistics was determined across the 500 simulated datasets.

These Monte Carlo simulations used a full factorial design in which the following factors were allowed to vary: $\beta_{0}$ (which will influence the overall probability of the condition occurring), $\exp\left(\beta_{1}\right)$, and $\sigma_{\text{mc}}$. We allowed $\beta_{0}$ to take on the values −2, −1, 0, 1, and 2; $\exp\left(\beta_{1}\right)$ to vary from 1 to 4 in increments of 0.2; and $\sigma_{\text{mc}}$ to vary from 0.2 to 4 in increments of 0.2. In each of the 1,600 (5 × 16 × 20) different scenarios, we computed: the mean of the empirical and predicted c-statistics and the mean of the skewness of the explanatory variable in those with and without the condition across the 500 simulated datasets that were generated under each scenario.

Data were simulated using the R statistical programming language [16]. The logistic regression models were fit using the *lrm* function in the *Design* package. The skewness of the explanatory variable was estimated using the *skewness* function in the *e1071* package.

### Monte Carlo simulations – results

**Figure 1:**
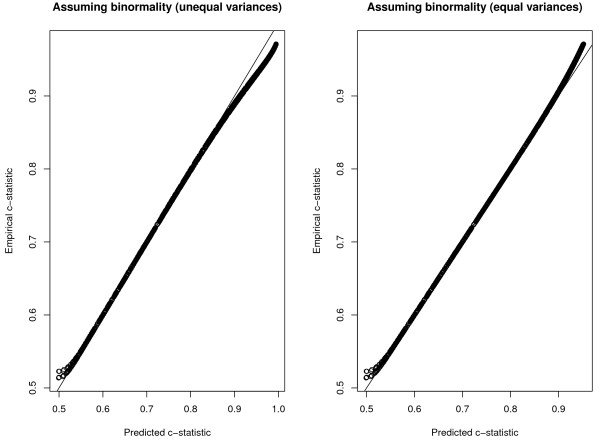
Comparison of empirical and predicted c-statistics: normal distribution.

The relationship between the predicted c-statistics and the empirical c-statistics across the scenarios is described in Figure 1. The left panel reports the predicted c-statistics when formula (1), which does not make the assumption of the equality of the variance of the explanatory variable in those with and without the condition, was used to predict the c-statistic. The right panel reports the predicted c-statistic when formula (2), which assumes that the variance of the explanatory variable was the same in those with and without the condition, was used. Both formulas provided very accurate prediction of the c-statistic when the predicted c-statistic was less than 0.80 to 0.90. Modestly more accurate predictions were obtained in the higher range of predicted c-statistics under the assumption of equality of variance.

**Figure 2:**
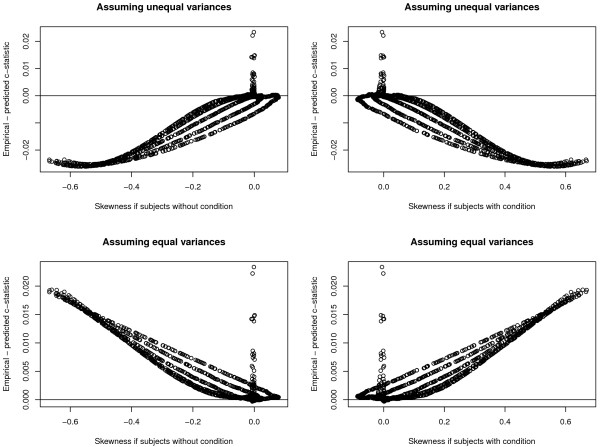
Difference between empirical and predicted c-statistic and skewness.

The relationships between the difference between the empirical and predicted c-statistics and the skewness of the distribution of the explanatory variable in those with and without the condition are reported in Figure 2. In general, one observes a pattern in which the difference between empirical and predicted c-statistics increased as the skewness of the distribution of the explanatory variable increased.

### Accuracy of predictions under non-normal distributions of the explanatory variable

The analytic derivations in Section 2 required the assumption of binormality. In Section 3, using an extensive set of Monte Carlo simulations, we found that the formulas derived under the assumption of binormality allowed for relatively accurate prediction of the empirical c-statistic when the distribution of the explanatory variable was normally distributed in the entire sample of those with and without the condition. In this section, we examine the accuracy of these predictions when the distribution of the explanatory variable was non-normal in the combined sample. We considered the following three distributions: gamma, log-normal, and uniform.

## Methods

We used Monte Carlo simulations similar to those described in Section 3. For each of the three non-normal distributions, we used a full factorial design. When the explanatory variable followed a gamma distribution, we allowed $\beta_{0}$ to take on the values −1, 0, and 1 and $\exp\left(\beta_{1}\right)$ to vary from 1 to 4 in increments of 0.25. The gamma distribution had the scale parameter fixed at 1, and the shape parameter was allow to vary from 0.25 to 4 in increments of 0.25. We thus considered 624 (3 × 13 × 16) different scenarios. When the explanatory variable followed a uniform distribution, we allowed $\beta_{0}$ to take on the values −2, −1, 0, 1, and 2, $\exp\left(\beta_{1}\right)$ to vary from 1 to 4 in increments of 0.2, and the parameter of the uniform distribution to vary from 0.2 to 4 in increments of 0.2 (the uniform distribution U(a) was parameterized so that its range was from –a to + a). We thus considered 1,600 (5 × 16 × 20) different scenarios. When the explanatory variable followed a log-normal distribution, we allowed $\beta_{0}$ to take on the values −1, 0, and 1, $\exp\left(\beta_{1}\right)$ to vary from 1 to 4 in increments of 0.25. The logarithm of the log-normal distribution had mean zero and its standard deviation varied from 0.1 to 2 in increments of 0.1. We thus considered 780 (3 × 13 × 20) different scenarios.

## Results

**Figure 3:**
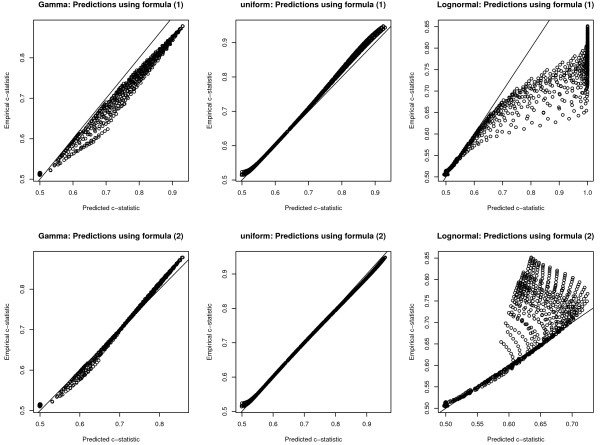
Comparison of empirical and predicted c-statistics: non-normal distributions.

The relationship between the empirical and predicted c-statistics is described in Figure 3. The top three panels describe the relationship when binormality with unequal variances (formula (1)) was used to predict the c-statistic. The lower three panels describe the relationship when binormality with equal variances (formula (2)) was used to predict the c-statistic. In general, better predictions were obtained using formula (2) compared to formula (1). Prediction was most accurate when the distribution of the explanatory variable was uniform, and was the least accurate when the distribution was log-normal. Prediction was relatively good when the explanatory variable followed a gamma distribution.

### Case study

We examined the ability of our derived formulas to predict the c-statistic for two logistic regression models in a sample of subjects hospitalized with acute myocardial infarction (AMI).

### Data sources

We used patients from the first phase of Enhanced Feedback for Effective Cardiac Treatment (EFFECT) Study, an initiative to improve the quality of care for patients with cardiovascular disease in Ontario [17,18]. Detailed clinical data were collected on patients hospitalized with AMI between April 1, 1999 and March 31, 2001 at 86 hospital corporations in Ontario, Canada, by retrospective chart review. Data on patient demographics, vital signs at presentation, medical history, and results of laboratory tests were collected for these patients. After excluding subjects with missing data on key variables, 9,298 subjects were available for use in this case study.

### Methods

The outcome of interest for the current example was whether the patient died within 30 days of hospitalization. We fit two different logistic regression models. The first was a univariate logistic regression model in which we regressed 30-day mortality on patient age. In the second model, we regressed mortality on the following baseline covariates: age, sex, cardiogenic shock, acute congestive heart failure/pulmonary edema, systolic blood pressure, diastolic blood pressure, heart rate, respiratory rate, diabetes, hypertension, current smoking status, dyslipidemia, family history of coronary artery disease, cerebrovascular disease/transient ischemic attack, angina, cancer, dementia, peptic ulcer disease, previous AMI, asthma, depression, peripheral vascular disease, previous revascularization, congestive heart failure, hyperthyroidism, aortic stenosis, haemoglobin, white blood count, sodium, potassium, glucose, urea, and creatinine.

We determined the empirical c-statistic for each of the two logistic regression models. We estimated the components of the distribution of age and of the linear predictor from the second logistic regression model that were necessary to predict the c-statistic using formulas (1) and (2). When using formula (2) with the multivariable model, we used β = 1, since the regression coefficient for the linear predictor would be one if the outcome were regressed on the linear predictor alone.

### Results

The empirical c-statistic of the univariate logistic regression model that regressed 30-day mortality on age was 0.759. The corresponding predicted c-statistics were 0.760 and 0.790 when formulas (1) and (2) were used, respectively. The empirical c-statistic of the multivariable model was 0.853. The corresponding predicted c-statistics were 0.849 and 0.855 when formulas (1) and (2) were used, respectively. The improved accuracy of prediction of the c-statistic for the multivariable model is likely due to the distribution of the linear predictor having a distribution that is closer to a normal distribution compared to the distribution of age.

## Discussion

Under the assumption that the explanatory variable was normally distributed in those with and without the condition, we derived an explicit expression for the c-statistic. We demonstrated that the c-statistic is a function of only the mean and variance of the explanatory variable in those with and without the condition. In particular, the c-statistic is a function of the standardized difference comparing the mean of the explanatory variable between those with and without the covariate. When the explanatory variable had the same variance in those with and without the condition, we demonstrated that the model c-statistic is an increasing function of the standard deviation of the normal distributions and of the log-odds ratio. The primary novelty of our findings is that the functional relationship of the c-statistic of a logistic regression model has now been described. Using an extensive set of Monte Carlo simulations, we found that our formulas provided reasonably accurate prediction when the distribution of the explanatory variable was normal in the entire sample of those with and without the condition. Some of our findings corroborate previous observations based on Monte Carlo simulations on how the c-statistic improved with increases in the odds ratio [19]. While our derivations are based on a single explanatory variable that is normally distributed in those with and without the condition, our results will generalize to any setting in which there is a real valued transformation of a set of explanatory variables, f(**X**), such that the distribution of f(**X**) is normal in those with and without the condition.

There are two implications of these findings for researchers constructing and interpreting predictive models for binary outcomes. First, it is widely known that greater discrimination is possible when the regression model contains independent explanatory variables that are strongly associated with the outcome [20]. However, we have demonstrated that when comparing the performance of the same regression model in different populations, a higher c-statistic is to be expected for the model fit in the population in which there is greater variation in the explanatory variable. Conversely, diminished predictive accuracy is to be expected in more homogeneous populations and samples, even if the odds ratio is transportable across populations. In a multivariable model, the linear predictor was recently suggested as the summary continuous variable to indicate population heterogeneity, with direct impact on the magnitude of the c-statistic [21]. In our case-study, we found that accurate prediction of the c-statistic was obtained from the distribution of the linear predictor. As noted above, the linear predictor is a real-valued function of the set of explanatory variables. Furthermore, the central limit theorem suggests that the distribution of the linear predictor will tend to be approximately normally distributed as both the sample size and the number of explanatory variables increases.

## Conclusions

In conclusion, when a continuous explanatory variable is normally distributed both in those with and without the outcome or condition, and these two normal distributions have equal variances, then the c-statistic follows a standard normal cumulative distribution function with dependence on the product of the standard deviation of the normal components (reflecting more heterogeneity) and the log-odds ratio (reflecting larger effects). When the explanatory variable is normally distributed in the combined population of subjects, then the formulas that we derived provide a reasonably accurate prediction of the empirical c-statistic. We conclude that discriminative ability of an explanatory variable cannot be judged by its odds ratio alone, but always needs to be considered in relation to the heterogeneity of the population.

## Competing interests

The authors declare that they have no competing interests.

## Authors' contributions

PA derived the mathematical relationships, conducted the Monte Carlo simulations, and drafted the initial version of the manuscript. ES provided insight on study design, interpretation, and revised the manuscript for important intellectual content. All authors read and approved the final manuscript.

## Pre-publication history

The pre-publication history for this paper can be accessed here:

http://www.biomedcentral.com/1471-2288/12/82/prepub
